# Correction: Thermodynamics of Random Reaction Networks

**DOI:** 10.1371/journal.pone.0124858

**Published:** 2015-04-08

**Authors:** The PLOS ONE Staff

During typesetting, errors were introduced into Fig 3, Fig 4 and Fig 5. In Fig 3, the legend text is missing, and the labeling of the y-axis is incomplete. In Fig 4, the “B” label for subfigure B is missing, the legend text in subfigure B is incomplete, and the labeling of the y-axis in subfigure A is incomplete. In Fig 5, most of the text is missing. Please view the complete, correct Fig 3, Fig 4 and Fig 5 below. The publisher apologizes for the errors.

**Fig 3:**
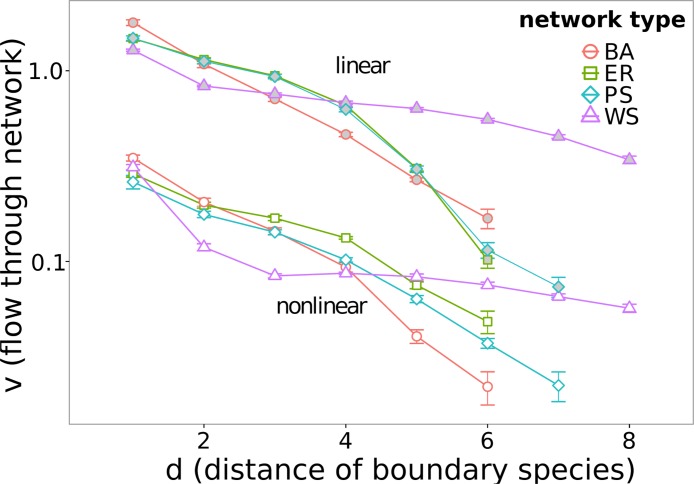
The flow *v* through the network depending on boundary species distance *d*. All networks are simulated with a boundary concentration difference of ∣*c*_1_−*c*_2_∣ = 0.9 and a base concentration of min(*c*_1_,*c*_2_) = 0.1. Filled (grey) symbols represent linear networks, empty (white) the nonlinear ones. Error bars show the standard error of the mean.

**Fig 4:**
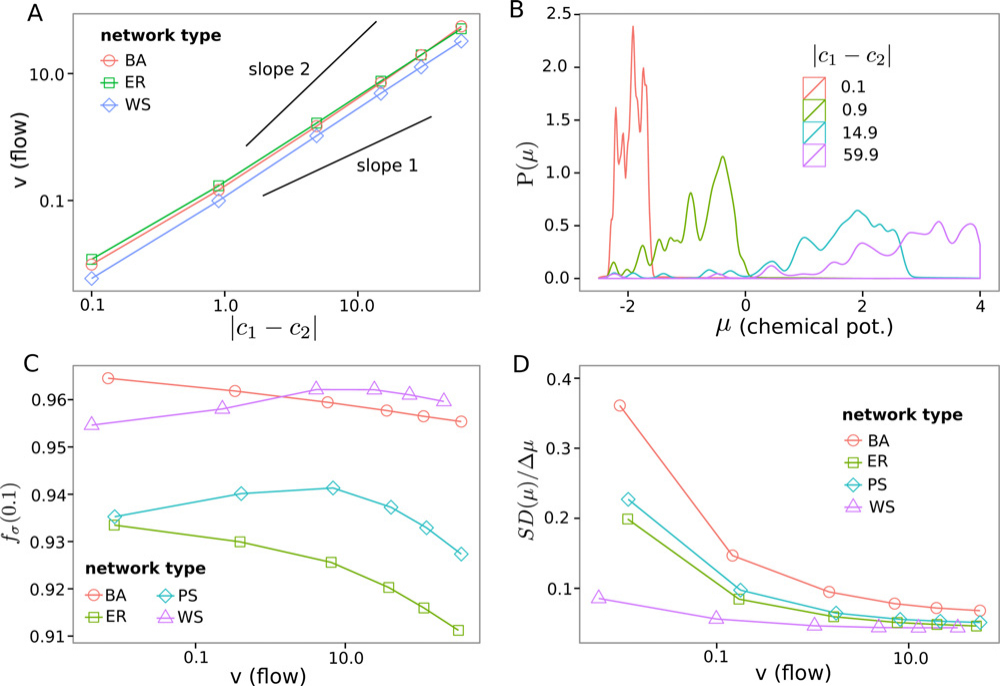
Varying flow through nonlinear networks. Each data point is the average of all simulations with specific boundary species concentration (*c*_1_ = 0.1 *c*_2_ = 0.2…60) and a shortest path between boundary species of 3. **(A)** Dependency of flow from concentration difference. Pan-Sinha results are not shown as they overlap with the Erdős-Rényi ones. **(B)** Distribution of species chemical potential *μ*_*i*_ for different boundary condition strengths of BarabsiAlbert (BA) networks. **(C)** The fraction of dissipation in the network explained by the most dissipating 10 percent of reactions, *f*_*σ*_(0.1). **(D)** Standard deviation of chemical potentials *σ*_*μ*_ normalized by difference between boundary species’ potentials Δ*μ* = ∣*μ*_*b*2_−*μ*_*b*1_∣ shows a more localized distribution of chemical potentials for larger flows.

**Fig 5:**
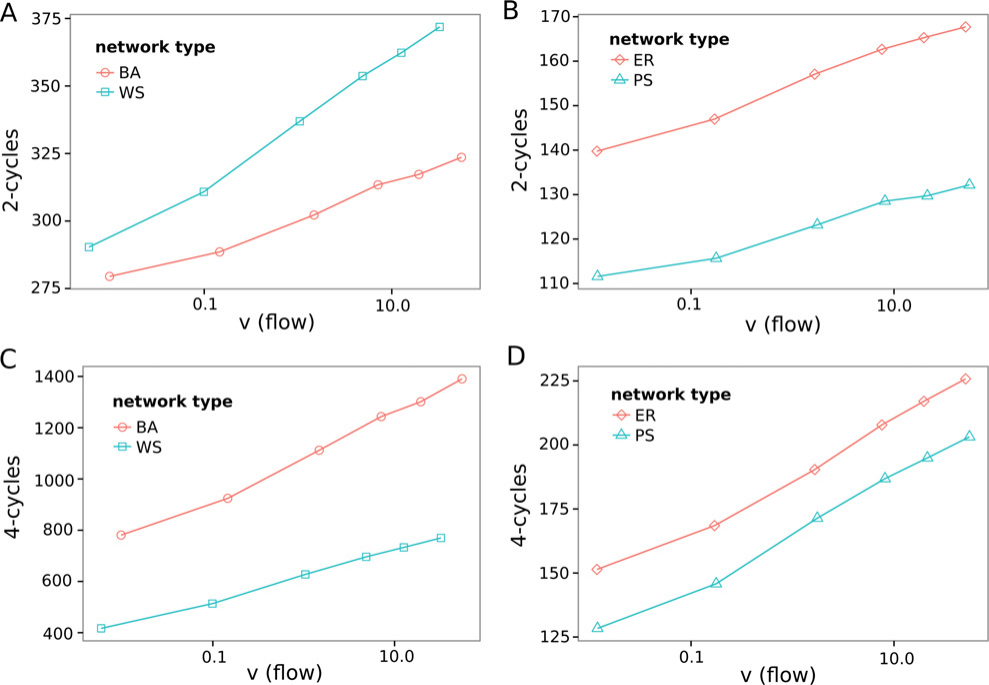
Number of 2- and 4-cycles in the (directed) substrate graphs of the nonlinear reaction networks. The plots show the number of additional cycles depending on the flow through the network in comparison to the same network with random reaction directions (Table 1). Each data point is the average of all simulations with boundary points distance of 3 and fixed boundary concentrations (*c*_1_ = 0.1 *c*_2_ = 0.2…60).
