# Correction: Thermodynamics of Random Reaction Networks

**DOI:** 10.1371/journal.pone.0128260

**Published:** 2015-05-08

**Authors:** The PLOS ONE Staff

## Notice of Republication

This article was republished on April 27, 2015, to correct errors in figures that were introduced during the typesetting process. The publisher apologizes for the errors. Please download this article again to view the correct version. The originally published, uncorrected article and the republished, corrected article are provided here for reference.

Please note that the republication addresses the issues in the correction posted on April 8, 2015.

## Supporting Information

S1 File Originally published, uncorrected article. (PDF)

S2 File Republished corrected article. (PDF)
