# Supplementary material for: Correction: Thermodynamics of Random Reaction Networks
Source: PLoS One. 2015 May 8;10(5):e0128260. doi: 10.1371/journal.pone.0128260 (PMC4425702; doi:10.1371/journal.pone.0128260)
Supplement: S1 File — (PDF) [file pone.0128260.s001.pdf]

PLOS | ONE

RESEARCH ARTICLE

Thermodynamics of Random Reaction Networks

Jakob Fischer[1,2,3]*, **Axel Kleidon**[2], **Peter Dittrich**[1]*

1 Bio Systems Analysis Group, Institute of Computer Science, Jena Centre for Bioinformatics and Friedrich Schiller University, Jena, Germany, **2** Max-Planck-Institute for Biogeochemistry, Jena, Germany, **3** International Max Planck Research School for Global Biogeochemical Cycles, Jena, Germany

* fischer.jakob@uni-jena.de (JF); peter.dittrich@uni-jena.de (PD)

Abstract

Reaction networks are useful for analyzing reaction systems occurring in chemistry, systems biology, or Earth system science. Despite the importance of thermodynamic disequilibrium for many of those systems, the general thermodynamic properties of reaction networks are poorly understood. To circumvent the problem of sparse thermodynamic data, we generate artificial reaction networks and investigate their non-equilibrium steady state for various boundary fluxes. We generate linear and nonlinear networks using four different complex network models (Erdős-Rényi, Barabási-Albert, Watts-Strogatz, Pan-Sinha) and compare their topological properties with real reaction networks. For similar boundary conditions the steady state flow through the linear networks is about one order of magnitude higher than the flow through comparable nonlinear networks. In all networks, the flow decreases with the distance between the inflow and outflow boundary species, with Watts-Strogatz networks showing a significantly smaller slope compared to the three other network types. The distribution of entropy production of the individual reactions inside the network follows a power law in the intermediate region with an exponent of circa −1.5 for linear and −1.66 for nonlinear networks. An elevated entropy production rate is found in reactions associated with weakly connected species. This effect is stronger in nonlinear networks than in the linear ones. Increasing the flow through the nonlinear networks also increases the number of cycles and leads to a narrower distribution of chemical potentials. We conclude that the relation between distribution of dissipation, network topology and strength of disequilibrium is nontrivial and can be studied systematically by artificial reaction networks.

CrossMark
click for updates

OPEN ACCESS

Citation: Fischer J, Kleidon A, Dittrich P (2015) Thermodynamics of Random Reaction Networks. PLoS ONE 10(2): e0117312. doi:10.1371/journal.pone.0117312

Academic Editor: Lev Tsimring, University of California San Diego, UNITED STATES

Received: July 4, 2014

Accepted: December 19, 2014

Published: February 27, 2015

Copyright: © 2015 Fischer et al. This is an open access article distributed under the terms of the Creative Commons Attribution License, which permits unrestricted use, distribution, and reproduction in any medium, provided the original author and source are credited.

Data Availability Statement: All relevant data are within the paper.

Funding: JF is funded by the International Max Planck Research School for Global Biogeochemical Cycles (http://www.imprs-gbgc.de/). The funders had no role in study design, data collection and analysis, decision to publish, or preparation of the manuscript.

Competing Interests: The authors have declared that no competing interests exist.

Introduction

Connecting network theory with thermodynamics was an idea already present more than 40 years ago under the term network thermodynamics [1]. Despite the fact that the terms were used in combination, the theory was merely a graphical representation of conservation equations and did not make any statements about complex networks, as they are known today. In 2006 Cantú and Nicolis [2] studied thermodynamic properties of linear networks, but limited

themselves to small networks, which they were able to handle analytically. Here, we extend their study by generating big random linear and nonlinear reaction networks and simulating them to a thermodynamically constrained steady state. This might contribute to a framework that allows to test methods for reconstructing thermodynamic data of reaction networks [3, 4] and lead to a better thermodynamic understanding of reaction networks in general. Possible applications of this approach include the thermodynamic investigation of reaction models in biology [3–5], origin of life [6] and also Earth system and planetary science [7, 8].

We look at reaction networks as thermodynamic systems that transforms two chemical species into one another [2]. The environment is driving the network to thermodynamic disequilibrium by keeping the concentration of two species constant. In the following, we will call the chemical species that are kept constant 'boundary species', because they are the species to which the boundary conditions are applied to.

Our basic assumption is that the network is able to transform the two boundary species into each other. This is not always possible in real reaction networks where the transformations are constrained by stoichiometry of chemical constituents. For example, any chemically sound reaction model will implicitly forbid pathways that transform N_2O into H_2O. Even if the artificial networks we create are comparable in density, they are not created with this constraint. This is due to the implications this constraint would have on the complexity of the boundary conditions. Omitting it leads to the existence of many transformation pathways between most pairs of randomly chosen boundary species, otherwise almost all pairs of boundary species would just have a steady state flow of zero between them.

We study different quantitative properties of the networks at steady state. In particular, because cycles have been reported to have important functions in networks [9–11], we look at the cycles that appear in the flow pattern. These cycles depend on the direction of the flow of each reaction, which in turn depends on the strength of the thermodynamic disequilibrium caused by the boundary condition.

In the next section we describe our method for generating reaction networks so they resemble different complex network models and how we simulate them to find their non-equilibrium steady state. We then present our results concerning the flow through the networks, the distribution of entropy production of individual reactions, and the dependency of cycle number from flow through the nonlinear networks.

Methods

Reaction Networks

Reaction networks [12] consist of a set of species $\mathcal{M}$ combined with a set of reactions $\mathcal{R}$. They contain information on the connection of chemical species through reactions and include the stoichiometric constraints given by the reactions. Mathematically, a reaction network can be described by two stoichiometric matrices **L** and **R**. L_{ij} is the coefficient of the i-th species on the left side of the j-th reaction and R_{ij} is the coefficient of the i-th species on the right side of the j-th reaction. Combining both matrices gives the stoichiometric matrix $\mathbf{N} = \mathbf{R} - \mathbf{L}$, for which the element N_{ij} in i-th row and j-th column gives the effective change of species i by reaction j. Given a relation $\mathbf{v} = \mathbf{v}(\mathbf{x})$ between reaction rates **v** and species concentrations **x**, one can associate the reaction network with the dynamics of an ordinary differential equation (ODE):

$$\frac{d\mathbf{x}}{dt} = \mathbf{N} \cdot \mathbf{v}(\mathbf{x}).$$

In complex network science instead of looking at a bipartite graph, where reactions and species are represented by different types of nodes which are connected by edges, often the

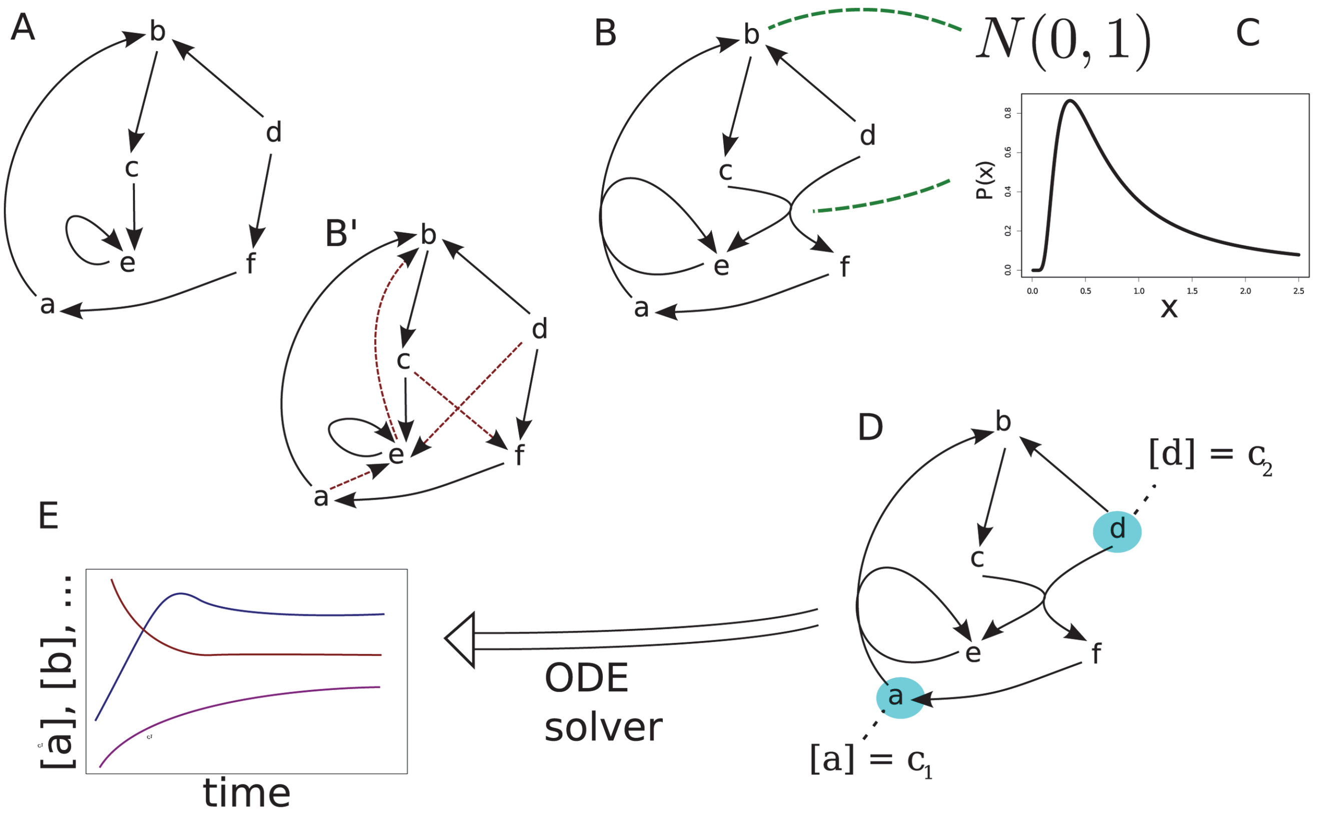

Fig 1. Illustration of realized simulations. (A) Linear reaction networks are generated from existing complex network models. (Arrows represent reactions, chemical species are indicated by lowercase letters.) **(B)** Pairs of linear reactions are combined to form nonlinear reactions. **(B')** Substrate graph that should maintain its characteristic properties while coupling. Edges invoked by coupling are depicted with dotted arrows. **(C)** Gibbs energies of formation are assigned to species from a normal distribution, activation energies to reactions from a Planck-like distribution (Eq. 8). **(D)** Two boundary species whose concentrations are kept constant are selected while the others are initialized randomly. **(E)** Reaction equation is solved numerically and final rates are taken as steady state rates.

doi:10.1371/journal.pone.0117312.g001

substrate graph is used. In this simplified view the nodes represent the species and an edge between two species is present if and only if there is a reaction having those two species on different sides of the reaction equation (Fig. 1, (B')) [13, 14].

Network Construction

Our artificial reaction networks are generated in three steps. We first generate a simple directed network (graph) consisting out of a set V of N nodes and a multiset $E \subset V \times V$ of M edges. These networks are generated following the models of Erdős-Rényi [15], Barabási-Albert [16] (scale-free), Watts-Strogatz [17] (small-world, clustering) and Pan-Sinha [18, 19] (hierarchically-modular). We are always using variants of these network models that allow formation of self loops and multiple edges between the same nodes. Also, we generate networks with a fixed number of edges. From these complex networks the reaction network is constructed.

Simple reaction networks are created by translating each edge into a reaction of the form $X \leftrightharpoons Y$ with X being the first and Y being the second node. In the rate equation of mass action kinetics this leads to a linear dependence of the reaction rates from the concentrations and thus we are calling these networks "linear" reaction networks.

Nonlinear reaction networks are generated out of directed networks by combining pairs of edges to second order reactions of the form $X+Z \leftrightharpoons Y+W$. The selection of pairs is done with a probability distribution that maintains the characteristic properties of the substrate graph as much as possible. This is done by considering the probability of newly introduced edges in the substrate graph in the originally used network model. For example, consider the combination of the reactions $A \leftrightharpoons B$ and $C \leftrightharpoons D$ to create the reaction $A+C \leftrightharpoons B+D$. This leads to two new edges in the substrate graph between A and D as well as between C and B. The probabilities of these two edges in the original network model are then used to calculate the probability of the combined reactions.

Finally, the thermodynamic data is generated and assigned to species and reactions. In the following, the generation process of nonlinear networks specific to the different network models is explained before the generation of thermodynamic data is specified in detail.

Erdős-Rényi (ER). In the Erdős-Rényi network model [15] all possible edges have the same probability. We create these networks by simply drawing the nodes of every edge from the set of all nodes with uniform probability. For the construction of nonlinear reaction networks, second order reaction equations are then chosen from the set of pairs of linear equations with uniform distribution. Note that linear equations that are used as part of a nonlinear equation are not returned leading to the probability of all pairs of linear equations containing it being set to zero for subsequent couplings.

Barabási-Albert (BA). For generating scale free networks, the Barabási-Albert models are used [16]. In this model nodes are added consecutively. Newly added nodes are connected to the network by introducing edges between it and already existing nodes. The selection of nodes to attach to is done with probability scaling with their node degree (preferential attachment).

The coupling probability of linear reactions is calculated from the product of the node degrees of the chemical products. In principle, other functional dependencies are possible, but for simplicity we choose this one and check that it maintains the power law distribution of the node degree in the associated substrate graph (Fig. 2 (A)).

Watts-Strogatz (WS). Networks having a comparable average path length to Erdős-Rényi but with a higher clustering are generated with the Watts-Strogatz model [17]. From a circular lattice like structure, a fraction α is randomly reordered. For the size of our networks we choose a value of $\alpha = 0.1$ (see Table 1).

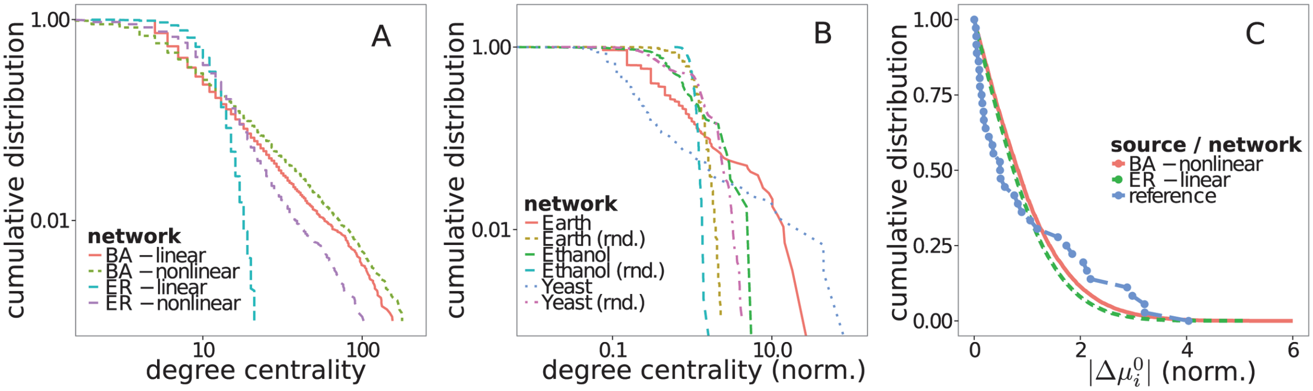

Fig 2. Comparison of artificial and real networks. (A) Nonlinear BA networks maintain scale-free degree distribution. **(B)** Cumulative degree scaling of real network's substrate graphs shows pronounced scale free property in comparison with their null models (randomized counterparts) for Earth's photochemistry [22] and a kinetik model of Yeast's metabolism [25]. **(C)** Cumulative distribution of the standard change of Gibbs energy of formation ($\Delta\mu_j^0 = \sum_i N_{ij}\mu_i^0$) for artificial networks and respective thermodynamic reference data for glycolysis (see [28], Table 4, $\Delta_r G'^0$). Distributions were (linearly) rescaled to have a mean of one.

doi:10.1371/journal.pone.0117312.g002

Table 1. Network properties. Properties of the substrate graphs of artificially generated networks as well as of examples of real networks. Table contains the number of vertices (|*V*|) and edges (|*E*|), the mean shortest path length ($< L >$) and the clustering coefficient ($< C >$) of the respective undirected network. The modularity is calculated using the walktrap community finding algorithm [38]. Data for real networks is taken from a database for Earth's photochemical reactions [22], models for the combustion of Ethanol [23] and Dimethyl ether [24] and a kinetic model of Yeast's metabolism [25]. For the artificial networks and the randomizations of the real networks mean values and standard deviations are calculated from 10 samples.

network	$\|V\|$	$\|E\|$	$< L >$	$< C >$	modularity	1-cycles	2-cycles	4-cycles
ER (linear)	1000	5000	4.5 ± 0.02	0.0098 ± 5e-04	0.234 ± 0.0037	4.6 ± 2.4	10 ± 2.3	151 ± 14
ER (nonlinear)	1000	5000	4.5 ± 0.03	0.0141 ± 0.002	0.296 ± 0.00921	4.1 ± 2	1634 ± 274	2964 ± 939
BA (linear)	1000	5000	3.9 ± 0.04	0.0277 ± 0.001	0.178 ± 0.00412	5.2 ± 2.4	47 ± 9.3	2138 ± 245
BA (nonlinear)	1000	5000	3.8 ± 0.04	0.0386 ± 0.002	0.266 ± 0.00664	9 ± 3.9	1254 ± 273	9321 ± 5306
WS (linear)	1000	5000	6.4 ± 0.1	0.484 ± 0.009	0.805 ± 0.00569	0.3 ± 0.48	2.4 ± 1.8	4188 ± 158
WS (nonlinear)	1000	5000	6.7 ± 0.1	0.255 ± 0.008	0.748 ± 0.00733	90 ± 5.1	1002 ± 58	2201 ± 161
PS (linear)	1000	5000	5 ± 0.03	0.0414 ± 0.003	0.51 ± 0.0124	469 ± 17	208 ± 24	316 ± 31
PS (nonlinear)	1000	5000	4.6 ± 0.06	0.0297 ± 0.001	0.314 ± 0.00817	285 ± 23	1782 ± 553	3758 ± 1745
Earth's atm.	280	1846	2.9	0.147	0.301	3	1337	48503
(randomized)	280	1846	3.3 ± 0.03	0.0513 ± 0.003	0.202 ± 0.011	6.2 ± 1.6	21 ± 5	480 ± 42
Ethanol	57	2902	1.7	0.4977	0.171	18	14264	1.59e+07
(randomized)	57	2902	1.4 ± 0.005	0.831 ± 0.006	0.051 ± 0.014	56 ± 7.4	1265 ± 22	1.49e+6 ± 19103
Dimethyl ether	79	2492	1.9	0.416	0.285	10	9995	3.45e+06
(randomized)	79	2492	1.7 ± 0.005	0.551 ± 0.007	0.073 ± 0.016	30 ± 4.9	492 ± 23	226961 ± 5134
Yeast Metab.	295	16954	2.6	0.1005	0.0175	27	355713	3.34e+09
(randomized)	295	16954	2 ± 0.01	0.51 ± 0.02	0.025 ± 0.045	59 ± 6.1	1715 ± 208	2.77e+06 ± 6.5e+05

doi:10.1371/journal.pone.0117312.t001

For the creation of nonlinear networks we only form those couplings between linear reactions which lead to two new close edges in the substrate graph. Here "close" means that their distance in terms of the circular lattice is not larger than the largest distance of non reordered edges in it. It would have be possible to use a more sophisticated approach and use the parameter α as the probability of introducing a far edge in the substrate graph while coupling. But because even our simple method does not achieve a clustering coefficient as high as equivalent linear networks (Table 1) we use this simple method.

Pan-Sinha (PS). Hierarchically-modular networks are generated starting with uniformly partitioning the nodes into 2^h elementary modules, with h being the number of hierarchical levels of the network. On the first level two pairs of modules on the elementary level are joined to form a new module, leading to $2^{(h-1)}$ modules on the first level. Analogous, for all other levels modules of the level below are joined pairwise, up to the h-th level where there is just one module consisting out of the entire network. When edges are added to the network this happens with a probability proportional to the lowest level l in which the two nodes to be connected share a module. Two nodes that share an elementary module are connected with the probability p_0 whose value is given by normalization. Nodes whose lowest common level is l are connected with probability $p_0\, p^l$. For our networks we choose $p = 0.5$ and $h = 8$.

When creating nonlinear reactions we assign each possible coupling a probability proportional to the product of the probability of the two newly introduced edges in the original model. Assuming a coupling leads to new edges in the substrate graph between nodes with lowest common module on levels l_1 and l_2, then the probability of choosing this coupling is scaled with $p^{l_1} p^{l_2}$.

Parameters. For network construction we generate linear reaction networks with $N =$ 1000 species and $M = 5000$ first-order reactions. Nonlinear networks are build by generating a linear network with $M = 3000$ reactions and connecting $C = 1000$ of them to second-order reactions. To compare linear and nonlinear networks directly we also generate linear networks

from the substrate graph of the nonlinear networks. This comparison is not possible with the generated linear reaction networks because their substrate graph is not as clustered. An overview of all generated networks is shown in Table 1.

Thermodynamics of Reaction Networks

Thermodynamic properties of reaction networks can be described by non-equilibrium thermodynamics [20, 21]. For simplicity and due to the artificial nature of our simulations we use unitless equations with the Boltzmann constant k_B and the temperature T set to one in this work (we do not consider variations in T).

The change of entropy dS can be separated into the exchange of the system with the environment $d_e S$ and change through processes in the system $d_i S$:

$$dS = d_e S + d_i S. \tag{1}$$

The entropy exchange with the environment (with constant temperature and pressure) is given by

$$d_e S = dU + pdV - \sum_k \mu_k d_e x_k, \tag{2}$$

with $d_e x_k$ being the change of concentration due to interaction with the environment and μ_k being the chemical potential. The entropy change through internal processes $d_i S$ is given accordingly:

$$d_i S = \sum_k \mu_k d_i x_k. \tag{3}$$

From this equation the rate of entropy production can be calculated. By rewriting we see that the entropy production of the network $\sigma_{tot} = \frac{d_i S}{dt}$ is merely the sum of the entropy production σ_i of all individual reactions with $\sigma_i = \sum_m \mu_m N_{mi} \nu_i$:

$$\frac{d_i S}{dt} = \sum_k \mu_k \frac{d_i x_k}{dt} = \sum_l \sum_m \mu_m N_{ml} \nu_l = \sum_l \sigma_l \tag{4}$$

In steady state the entropy production of individual reaction i can be also written as function of forward and backward reaction rates $\nu_{i,+}$ and $\nu_{i,-}$[21]:

$$\sigma_i = (\nu_{i,+} - \nu_{i,t-}) \ln \frac{\nu_{i,+}}{\nu_{i,-}}. \tag{5}$$

This relation can be applied to calculate the total entropy production rate $\sigma_{tot} = \sum_i \sigma_i$ of a reaction network acting between two boundary species b_1 and b_2 kept at concentrations c_1 and c_2. As the entropy production rate in steady state only depends on the boundary conditions (c_1,c_2,$\nu = \nu_+ - \nu_-$) we can replace the entire network with one imaginary linear reaction $b_1 \leftrightharpoons b_2$. If we assume the Gibbs energies of formation of boundary species to be zero, the forward and backward rate coefficients are equal and we obtain the equation

$$\sigma_{tot} = \nu \ \ln \frac{c_1}{c_2}. \tag{6}$$

Alternatively one could also get this result by calculating the boundary species entropy exchange with the environment, because in steady state $0 = dS = d_e S + d_i S$.

Generating Thermodynamic Data. The Gibbs energies of formation of the species μ_i^0 are drawn from a normal distribution $N(0,1)$. Reaction rates are calculated using the Arrhenius

equation (with the prefactor A set to 1):

$$k = Ae^{-E_a} = e^{-E_a}. \tag{7}$$

Here, E_a is the activation energy which is sampled from the distribution

$$P(x) = \frac{6}{\pi^2} \frac{1}{x^3(\exp(1/x) - 1)} \tag{8}$$

for every reaction. We have chosen this distribution, which resembles the Planck-distribution, because it has an effective non-zero lower bound while still having a large tail to the right (Fig. 1). We simulate all reactions reversibly. Forward and backward reaction are energetically constrained by the Gibbs energies of the species. Thus, we sample E_a just once for every reaction and assign it to that reaction direction which respective products have a higher Gibbs energy of formation, either $E_{e,i} = \sum_{j,N_{ij}>0} \mu_i^0 N_{ij}$ (forward direction) or $E_{p,i} = \sum_{j,N_{ij}<0} \mu_i^0 N_{ij}$ (backward direction). The second reaction directions activation energy is then given by the constraint $E'_e = E_e + |\sum_j \mu_i^0 N_{ij}|$. This expression is a reflection of the fact that in equilibrium, forward and backward reaction rates need to balance.

Network Simulation

As we are interested in the steady state of the network under thermodynamic boundary conditions we solve the reaction equation while keeping the concentration of two selected chemical species b_1, b_2 at fixed concentration c_1, c_2. To remove the effects of the energy difference between the boundary species on the flow, we set their Gibbs energy of formation μ_i^0 to zero and recalculate reaction rates before the simulation. For solving the reactions' ODE, the integrator of C++'s boost library is used. The selected algorithm is "Dormand-Prince 5". Concentrations are initialized normally distributed with $\frac{c_1+c_2}{2}$ taken as mean and $|c_1-c_2|$ as standard deviation. Dynamics are simulated up to a time t of 50000 or up to the time when the mean square change of concentration (per species and time-step size) is smaller than 10^{-20}.

We assume that the greatest topological factor influencing flow through the reaction network is the shortest path distance between the boundary species. Because we cannot perform simulation and analysis for one million pairs of boundary species, we sample 50 pairs of boundary species for all values of the shortest path occurring in the network.

A simple investigation of network flow and entropy distribution is done with boundary species concentrations set to $c_1 = 0.1$ and $c_2 = 1$. To get an error estimate, we generate 10 independent samples of every network type.

To investigate the response of the nonlinear networks to an increase in thermodynamic disequilibrium we vary the boundary conditions accordingly. For this we keep c_1 at 0.1 while varying c_2 from 0.2 up to 60. With higher values of boundary concentrations we notice an extreme increase of computational time needed to solve the individual ODEs. Thus, we are only able to simulate one network sample of every type for this setup.

The software for generating (https://github.com/jakob-fischer/jrnf_tools), running (https://github.com/jakob-fischer/jrnf_int), and analyzing (https://github.com/jakob-fischer/jrnf_R_tools) the simulations is developed in R and C++ and freely available through the platform github.

Results

For our study we generate various random networks for each network model (Table 1). We simulate these networks for different boundary conditions and analyze the resulting steady

state. In the following, we first compare the artificial networks with real networks and then show in detail how the flow and and energy dissipation depend on network structure and boundary condition.

Network Structure

We compare the topological features of our artificially generated networks with real world networks (Table 1). For this we use a compilation of chemical reactions in Earth's atmosphere [22] and models for the combustion of Methane [23] and Dimethyl ether [24]. Also a kinetic model of the metabolic network of Yeast [25], available through the BioModels Database [26], is investigated. To avoid that the representation of networks as substrate graphs biases our results [27] we compare each network with a randomized version of itself. When randomizing an artificial network we would obtain an Erdős-Rényi network with the same density and the same types of reactions. Thus, rows for randomized BA, WS, and PS networks are omitted in Table 1.

The power law scaling for Earth's atmospheric reaction network and the metabolic network of Yeast are clearly pronounced in comparison with their respective null models (Fig. 2 (B)). This is not true for the Ethanol combustion chemistry whose size of 57 species (nodes) does not allow to unambiguously decide on the scale-free property. The substrate graphs of the two combustion chemistries show the properties of small world networks, they have a small mean shortest path length and a high clustering coefficient. As their null models show the same properties, this can be attributed to their high density. All reaction networks have more cycles than their randomized counterparts. With the exception of the network from Yeast's metabolism all real networks also have a higher clustering coefficient or a higher value for modularity.

For a comparison of the artificial reaction networks with real thermodynamic data we use a table of reaction free energies ($\Delta_r G^0$) of reactions in glycolysis [28]. In our networks this corresponds to $\Delta\mu_j^0 = \sum_j N_{ij}\mu_i^0$. Because there is no way to assign a unique reaction direction to the reactions in the artificial networks, we are only comparing the distributions of absolute values $|\mu_j^0|$. The normalized (mean set to one) cumulative distributions show a more localized distribution with a wider tail for the data from glycolysis. The distribution for the artificial networks is over all more regular. The bimodal distribution for the data from glycolysys might be related to the fact that it describes two distinct processes, the tricarboxylic acid cycle and the pentose phosphate pathway.

Distance Dependency of Flow

To characterize the strength of the steady state flow for different network types, we start with the intuitive assumption that the main factor determining the flow is the distance between the two boundary species in the reaction network, measured by shortest path length d in the substrate graph. The dependency of the mean flow on shortest path length is shown in Fig. 3.

The flow through reaction networks created with small-world and clustering topology (Watts-Strogatz model) shows to be especially weakly dependent on boundary species distance d. In the linear as well as the nonlinear case these networks have a lower mean flow for small d (≤ 4) while for larger values of d, they have generally a larger flow than the other networks. We hypothesize that the flow for boundary points whose distance is close to the diameter is limited by the sparse connection of those boundary species to the network. The high clustering of Watts-Strogatz networks (cf. Table 1) apparently leads to their exceptional high flow for boundary points with a large distance d. This also agrees with the low sensitivity to boundary species distance that the Watts-Strogatz networks show.

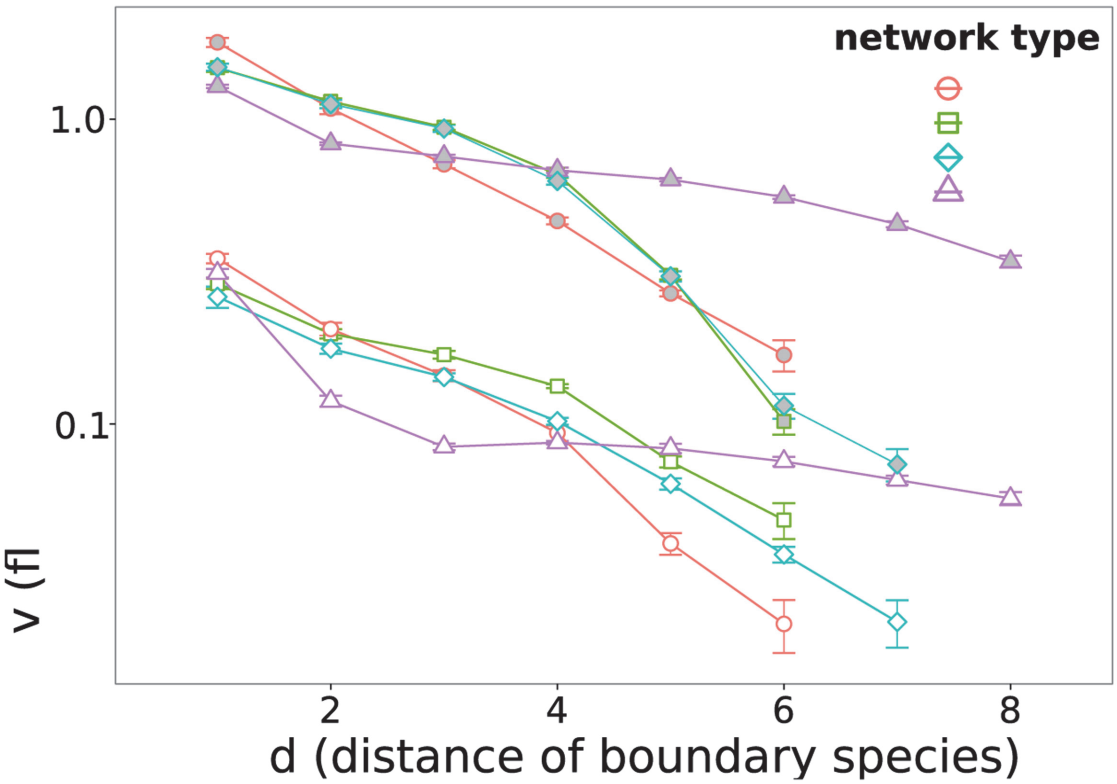

Fig 3. The flow *v* through the network depending on boundary species distance *d*. All networks are simulated with a boundary concentration difference of $|c_1 - c_2| = 0.9$ and a base concentration of $\min(c_1, c_2) = 0.1$. Filled (grey) symbols represent linear networks, empty (white) the nonlinear ones. Error bars show the standard error of the mean.

doi:10.1371/journal.pone.0117312.g003

The linear networks generated out of the Erdős-Rényi model and those generated with the Pan-Sinha model show a strikingly similar behavior. This may be due to their similar degree distribution (not shown).

Varying Flow through Nonlinear Networks

Unlike in linear networks, the flow and dissipation distribution in nonlinear networks depend on the absolute concentrations of the boundary species. For the variation of boundary concentration flow dependency of the concentration difference is in an intermediate regime (Fig. 4 (A)) and the slope in log-log plot takes a value between 1 and 2. This is plausible since the network consists of a mix of linear reactions and nonlinear reactions with at best quadratic behavior. Theoretically a stronger than quadratic dependency of flow from concentration difference would be possible for a specific boundary condition and a specific concentration range, but this possibility seems not to influence the mean behavior.

We look at the distribution of chemical potentials $\mu = \mu_0 + \ln(x_i)$ inside the reaction network for different strengths of the boundary condition. In Fig. 4 (B) the distributions $P(\mu)$ are shown for the simulated Barabási-Albert (BA) reaction networks with boundary species distance d of 3. The distributions in general are localized between the chemical potentials of the boundary species $\mu_{b_1} = \ln(x_{b_1})$ and $\mu_{b_2} = \ln(x_{b_2})$ (remember that μ_0 for boundary species is set to zero). While the distributions are almost uniform in this range for low flows, at higher flows the distributions are more shifted towards the upper part. Normalizing the standard deviation σ_μ by

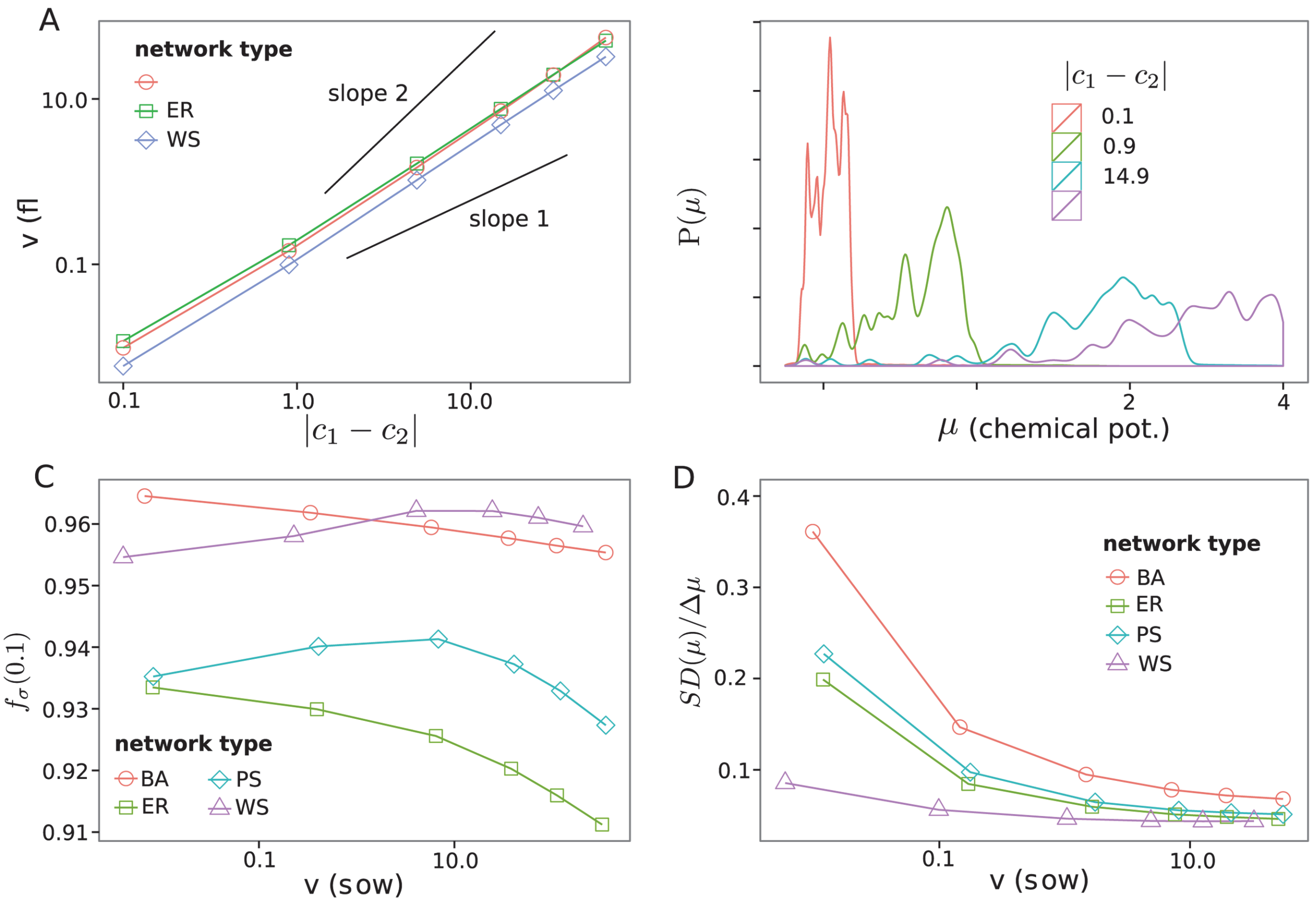

Fig 4. Varying flow through nonlinear networks. Each data point is the average of all simulations with specific boundary species concentration ($c_1 = 0.1$ $c_2 = 0.2...60$) and a shortest path between boundary species of 3. **(A)** Dependency of flow from concentration difference. Pan-Sinha results are not shown as they overlap with the Erdős-Rényi ones. **(B)** Distribution of species chemical potential μ_i for different boundary condition strengths of BarabsiAlbert (BA) networks. **(C)** The fraction of dissipation in the network explained by the most dissipating 10 percent of reactions, $f_\sigma(0.1)$. **(D)** Standard deviation of chemical potentials σ_μ normalized by difference between boundary species' potentials $\Delta\mu = |\mu_{b_2} - \mu_{b_1}|$ shows a more localized distribution of chemical potentials for larger flows.

doi:10.1371/journal.pone.0117312.g004

$\Delta\mu = |\mu_{b_2} - \mu_{b_1}|$ confirms this finding (Fig. 4 (D)) and shows a narrower distribution relative to the chemical potentials of the boundary species.

The distributions of dissipation values of the reactions are to noisy to find out if they also get narrower for higher flows. Thus, we calculate the fraction of the dissipation explained by the 10% of reactions with the highest dissipation, $f_\sigma(0.1)$. We see that with higher flows the fraction of dissipation explained by these 10 percent of the network decreases (Fig. 4 (C)). The networks generated from the Watts-Strogatz (WS) and the Pan-Sinha (PS) networks show an increase of $f_\sigma(0.1)$ for lower values, but above a flow of around 5 they also decrease. Explained differently, for higher flows one needs a larger part of the network to explain a given fraction of its dissipation. Together with the narrower distribution of chemical potentials we interpret this as the thermodynamic disequilibrium leading to a tighter coupling of the reaction network. This coupling leads to the chemical potential of different species to be closer and to the dissipation being more evenly distributed among reactions.

Flow Dependency of Cycle Number in Nonlinear Networks

There are many indicators that cycles have an important function in networks [9–11]. Cycles function as feedback mechanisms and stabilize the dynamics of the system against perturbations. Also cyclicity has been related to thermodynamic efficiency [29]. To check if there is a dependency of the number of cycles on the flow through the networks we count the number of small cycles (2- and 4-cycles) in the directed substrate graph for different values of ν. Note, that even if the simulated reactions do not change, a change in the effective flow of a reaction can imply a change of direction and by this a change in the directed substrate graph.

The number of small cycles is dependent on local topological properties of the network models. Thus, for evaluation we subtract the number of cycles found in networks with randomly chosen reaction directions (Table 1). For all network types we find a clear increase in the number of cycles with increasing flow (Fig. 5). This formation of additional cycles can be understood as the network self-organizes in thermodynamic disequilibrium to increase its flow and dynamic stability. Note that this supports the idea of the previous section of a closer coupling of the network with higher degree of disequilibrium.

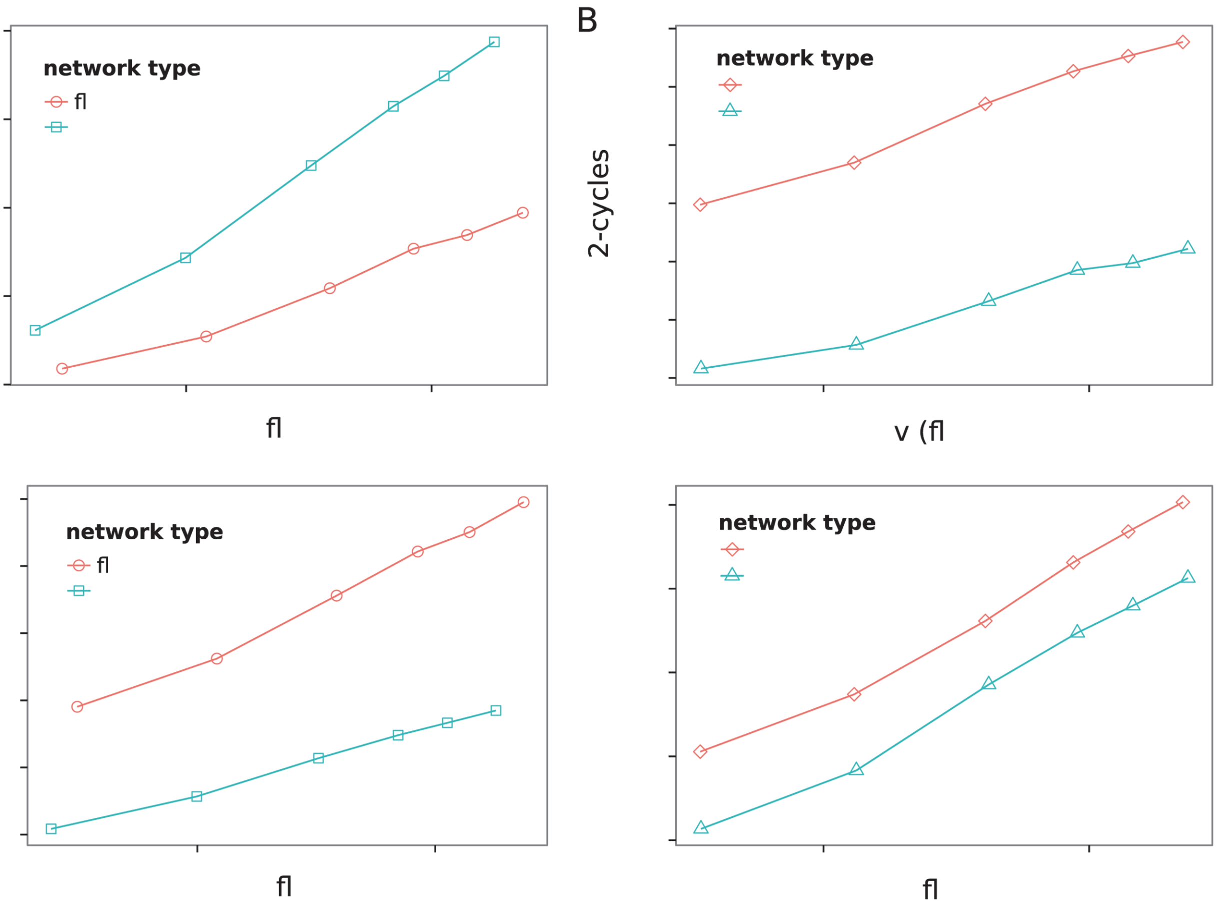

Fig 5. Number of 2- and 4-cycles in the (directed) substrate graphs of the nonlinear reaction networks. The plots show the number of additional cycles depending on the flow through the network in comparison to the same network with random reaction directions (Table 1). Each data point is the average of all simulations with boundary points distance of 3 and fixed boundary concentrations ($c_1 = 0.1$ $c_2 = 0.2...60$).

doi:10.1371/journal.pone.0117312.g005

Distribution of Entropy Production Rates

To see how dissipation is distributed inside of the networks, we calculate the entropy production rate for the individual reactions σ (Eq. 5) and look at their distribution for specific network topologies and boundary conditions. To better see the power law dependency, we plot the cumulative distribution $1 - \int_{-\infty}^{\sigma} P(\sigma')d\sigma'$, which describes the probability of the entropy production rate being higher than σ[30] instead of $P(\sigma)$.

The distributions show no large qualitative differences between the different network models (Fig. 6). The power law in the intermediate regime is differently pronounced in its extent for different network types but the greatest difference is clearly seen between the slopes of linear and nonlinear networks. Assuming that $P(\sigma)$ follows a power law, we get an exponent of about -1.5 for linear networks and of -1.66 for nonlinear networks. The steeper slope of the nonlinear networks can be interpreted as an effect of their reactions being better coupled. This can be seen by the fact that nonlinear ($A+B \leftrightharpoons C+D$) reactions are not depleting a potential between two species directly but there is always the probability that they increase the potential between two other species. The coupling implies a stronger connection of the flow between individual reactions and by this a stronger connection with the magnitude of dissipation.

Connectivity Dependence of Dissipation

To evaluate how the dissipation of a reaction depends on the connectivity of the involved species, for every species we calculate the mean dissipation of all reactions connected to it. Plotting the mean dissipation depending on the degree centrality of the species (in the substrate graph) shows a relatively high dissipation for reactions adjacent to lowly connected species (Fig. 7). This effect is more pronounced for nonlinear networks. When looking for reactions with high dissipation we should search in the vicinity of lowly connected species. This can be explained by the stronger connection between reactions generating and consuming the species. When the rate of a reaction that produces a species is increased, the additional flow has to be distributed

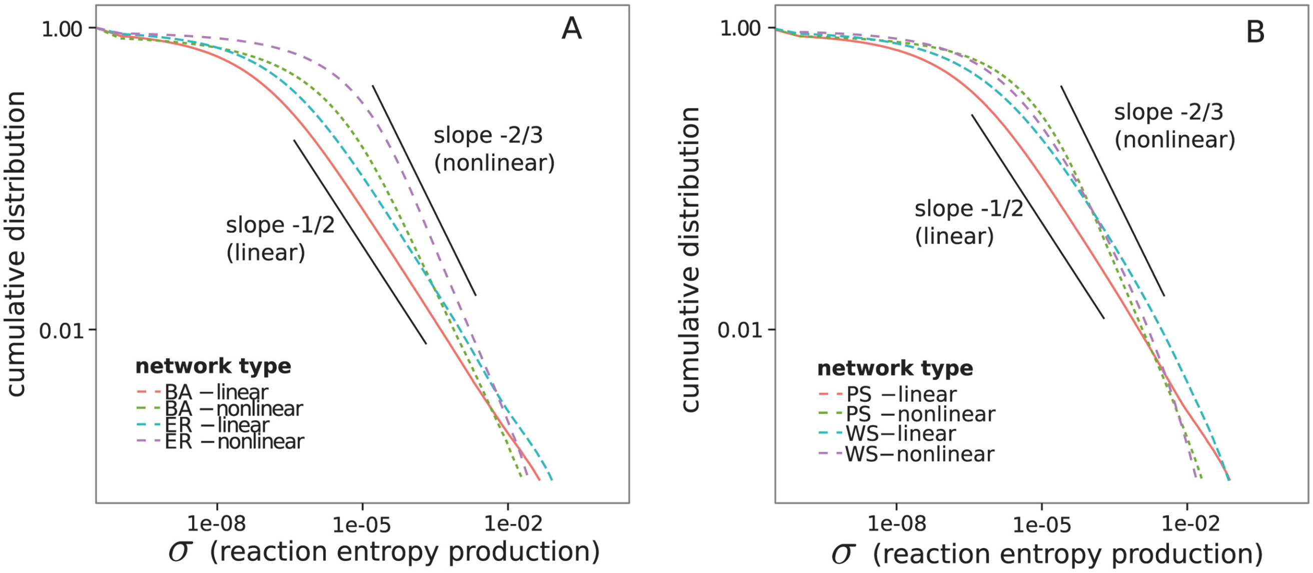

Fig 6. Cumulative distribution of the entropy production of the reactions. All simulations are performed with boundary concentration values of $c_1 = 0.1$, $c_2 = 1.0$ and a shortest path between boundary species of length 4. **(A)** Distributions for Barabási-Albert (BA) and Erdős-Rényi (ER) networks. **(B)** Distributions for Watts-Strogatz (WS) and Pan-Sinha (PS) networks.

doi:10.1371/journal.pone.0117312.g006

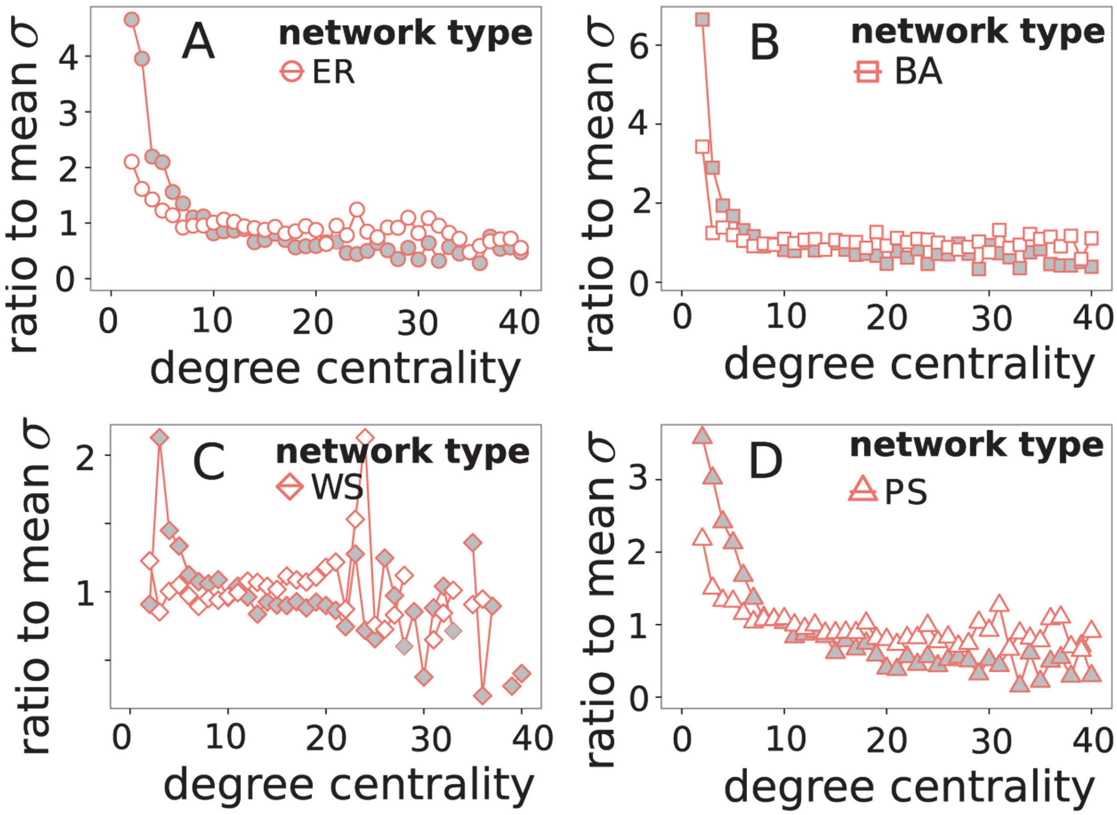

Fig 7. Mean entropy production σ associated with nodes of degree f. Values are normalized by mean entropy production in the sample network. Grey filled points show nonlinear networks, white filled points show linear networks. Data was taken from all simulation runs of the specified network type with min $(c_1,c_2) = 0.1$, $|c_1-c_2| = 0.9$ and shortest path $d = 4$.

doi:10.1371/journal.pone.0117312.g007

over the consuming reactions. If there are many consuming reactions, there are more potential pathways to forward the flow while keeping the mean dissipation rate low.

Conclusions

We have simulated random reaction networks under thermodynamic constraints in order to provide insight into how energy is dissipated in complex reaction networks in thermodynamic disequilibrium. We observe a clear difference between linear and nonlinear networks. The power law has an exponent of ≈ -1.5 for linear and a slightly lower exponent of ≈ -1.66 for nonlinear networks. However, there are no qualitative differences between the distributions of entropy production rate for different complex network models like Erdős-Rényi, Barabási-Albert, etc. (Fig. 6). The differences between thermodynamic reaction networks of different topologies are more pronounced in the flow (Fig. 3) than in the other properties investigated.

We found that a greater disequilibrium in nonlinear reaction networks is associated with a more tightly coupled network. For a greater flow, the network self-organizes and maintains a greater number of cycles (Fig. 5). A greater flow also leads to a narrower distribution of chemical potentials (Fig. 4 (B), (D)). This is associated with results that suggest that for higher flows, a larger fraction of the network is necessary to explain a given fraction of its dissipation (Fig. 4 (C)). We interpret this as an increase in the system's complexity that comes along with a higher thermodynamic disequilibrium.

Finally, we found that reactions involving lowly connected species tend to dissipate more energy, which is more pronounced in nonlinear networks, but is also found in linear networks (Fig. 7). This might help to identify reactions that play central roles in the energy dissipation of a complex reaction network.

We also showed how our artificial networks share topological properties with real reaction networks. The artificial networks are toplogically more similar to Earth's atmospheric chemistry and Yeast's metabolism than to the two investigated combustion chemistries. The main discriminating factor here is the high density of those two combustion chemistries. The distribution of thermodynamic parameters in the artificial networks only roughly matches data from reactions of glycolysis (Fig. 2 (B)). Obviously, the amount of thermodynamic data (37 reactions) is quite limited. Current progress in bioinformatic methods to reconstruct thermodynamic data [31–33] may improve the availability of such data in future and allow a better analysis.

Nevertheless, a fundamental problem of such a comparison remains. It is the way the data of reaction networks is obtained. In networks from chemical models, experimentalists and modelers have made a decision on which reactions are relevant. Experimentalists only find reactions that are occurring and are measurable in the systems they investigate. Also the modelers might just decide to exclude reactions with low reaction rates from their models. Hence, the reaction network taken from a model is already biased with respect to the model's intention. Our approach with artificial networks, however, assumes the artificial network is a set of (hypothetically) possible reactions; which reactions become important emerges from the dynamics and can be different depending on the boundary conditions.

Thus, we suggest to investigate such emergent phenomena in the future. This is possible by taking smaller artificial reaction networks and then looking at their reaction pathways using elementary flux modes [34]. This would also allow to test the relationship between the rate of an elementary mode and its entropy production [35, 36]. The thermodynamics of reaction networks and of cycling processes therein may also provide insight into the origins of life. Revealing how thermodynamics constraints the behavior of complex reaction networks will be an important ingredient in understanding the role of thermodynamics in domains like prebiotic chemistry [11, 37], biogeochemistry, and cellular systems.

Author Contributions

Conceived and designed the experiments: JF AK PD. Performed the experiments: JF. Analyzed the data: JF AK PD. Contributed reagents/materials/analysis tools: JF. Wrote the paper: JF AK PD.

References

1. Oster G, Perelson A, Katchalsky A (1971) Network thermodynamics. Nature 234: 393–399. doi: 10.1038/234393a0
2. Cantú AG, Nicolis G (2006) Toward a Thermodynamic Characterization of Chemical Reaction Networks. J Non-Equil Thermodyn 31: 23–46.
3. Soh KC, Hatzimanikatis V (2010) Network thermodynamics in the post-genomic era. Curr Opin Microbiol 13: 350–357. doi: 10.1016/j.mib.2010.03.001 PMID: 20378394
4. Soh KC, Miskovic L, Hatzimanikatis V (2012) From network models to network responses: integration of thermodynamic and kinetic properties of yeast genome-scale metabolic networks. FEMS Yeast Res 12: 129–143. doi: 10.1111/j.1567-1364.2011.00771.x PMID: 22129227
5. Martínez VS, Quek LE, Nielsen LK (2014) Network thermodynamic curation of human and yeast genome-scale metabolic models. Biophys J 107: 493–503. doi: 10.1016/j.bpj.2014.05.029 PMID: 25028891

6. Hordijk W, Steel M (2012) Autocatalytic sets extended: Dynamics, inhibition, and a generalization. J Syst Chem 3: 1–12. doi: 10.1186/1759-2208-3-5
7. Kasting JF, Holland HD, Pinto JP (1985) Oxidant abundances in rainwater and the evolution of atmospheric oxygen. J Geophys Res-Atmos 90: 497–510. doi: 10.1029/JD090iD06p10497
8. Saunders SM, Jenkin ME, Derwent RG, Pilling MJ (2003) Protocol for the development of the master chemical mechanism, mcm v3 (part a): tropospheric degradation of non-aromatic volatile organic compounds. Atmos Chem Phys 3: 161–180. doi: 10.5194/acp-3-161-2003
9. Gleiss PM, Stadler PF, Wagner A, Fell DA (2001) Relevant cycles in chemical reaction networks. Adv Complex Syst 4: 207–226. doi: 10.1142/S0219525901000140
10. Kreyssig P, Escuela G, Reynaert B, Veloz T, Ibrahim B, et al. (2012) Cycles and the qualitative evolution of chemical systems. PLOS ONE 7: e45772. doi: 10.1371/journal.pone.0045772 PMID: 23071525
11. Pascal R, Pross A, Sutherland JD (2013) Towards an evolutionary theory of the origin of life based on kinetics and thermodynamics. Open Biol 3: 130–156. doi: 10.1098/rsob.130156
12. Clarke BL (1988) Stoichiometric network analysis. Cell Biochem Biophys 12: 237–253.
13. Solé RV, Munteanu A (2004) The large-scale organization of chemical reaction networks in astrophysics. Europhys Lett 3: 1–7.
14. Wagner A, Fell DA (2001) The small world inside large metabolic networks. P Roy Soc Lond B Bio 268: 1803–1810. doi: 10.1098/rspb.2001.1711
15. Erdős P, Rényi A (1959) On random graphs i. Publ Math Debrecen 6: 290–297.
16. Albert R, Barabási AL (2002) Statistical mechanics of complex networks. Rev Mod Phys 74: 47–97. doi: 10.1103/RevModPhys.74.47
17. Watts DJ, Strogatz SH (1998) Collective dynamics of 'small-world' networks. Nature 393: 440–442. doi: 10.1038/30918 PMID: 9623998
18. Pan RK, Sinha S (2009) Modular networks with hierarchical organization: The dynamical implications of complex structure. Pramana 71: 331–340. doi: 10.1007/s12043-008-0166-1
19. Pan RK, Sinha S (2007) Modular networks emerge from multiconstraint optimization. Phys Rev E 76: 1–4. doi: 10.1103/PhysRevE.76.045103
20. Kschischo M (2010) A gentle introduction to the thermodynamics of biochemical stoichiometric networks in steady state. Eur Phys J Special Topics 187: 225–274. doi: 10.1140/epjst/e2010-01290-3
21. Kondepudi D, Prigogine I (1998) Modern thermodynamics / from heat engines to dissipative structures. Chichester; Weinheim: Wiley.
22. Yung YL, DeMore WB (1998) Photochemistry of Planetary Atmospheres. New York: Oxford University Press.
23. Marinov NM (1999) A detailed chemical kinetic model for high temperature ethanol oxidation. Int J Chem Kinet 31: 183–220. doi: 10.1002/(SICI)1097-4601(1999)31:3%3C183::AID-KIN3%3E3.0.CO;2-X
24. Kaiser EW, Wallington TJ, Hurley MD, Platz J, Curran HJ, et al. (2000) Experimental and modeling study of premixed atmospheric-pressure dimethyl ether-air flames. J Phys Chem A 104: 8194–8206. doi: 10.1021/jp994074c
25. Stanford NJ, Lubitz T, Smallbone K, Klipp E, Mendes P, et al. (2013) Systematic construction of kinetic models from genome-scale metabolic networks. PLOS ONE 8: e79195. doi: 10.1371/journal.pone.0079195 PMID: 24324546
26. Li C, Donizelli M, Rodriguez N, Dharuri H, Endler L, et al. (2010) Biomodels database: An enhanced, curated and annotated resource for published quantitative kinetic models. BMC Syst Biol 4: 92. doi: 10.1186/1752-0509-4-92 PMID: 20587024
27. Zhou W, Nakhleh L (2011) Properties of metabolic graphs: biological organization or representation artifacts? BMC Bioinformatics 12: 132. doi: 10.1186/1471-2105-12-132 PMID: 21542923
28. Li X, Wu F, Qi F, Beard DA (2011) A database of thermodynamic properties of the reactions of glycolysis, the tricarboxylic acid cycle, and the pentose phosphate pathway. Database 2011: bar005.
29. Layton A, Reap J, Bras B, Weissburg M (2012) Correlation between thermodynamic efficiency and ecological cyclicity for thermodynamic power cycles. PLOS ONE 7: e51841. doi: 10.1371/journal.pone.0051841 PMID: 23251638
30. Newman MEJ (2005) Power laws, pareto distributions and zipf's law. Contemp Phys 46: 323–351. doi: 10.1080/00107510500052444
31. Cannon WR (2014) Simulating metabolism with statistical thermodynamics. PLOS ONE 9: e103582. doi: 10.1371/journal.pone.0103582 PMID: 25089525

32. Chakrabarti A, Miskovic L, Soh KC, Hatzimanikatis V (2013) Towards kinetic modeling of genome-scale metabolic networks without sacrificing stoichiometric, thermodynamic and physiological constraints. Biotechnol J 8: 1043'1057. doi: 10.1002/biot.201300091 PMID: 23868566
33. De Martino D (2013) Thermodynamics of biochemical networks and duality theorems. Phys Rev E 87: 052108. doi: 10.1103/PhysRevE.87.052108
34. Schuster R, Schuster S (1993) Refined algorithm and computer program for calculating all non–negative fluxes admissible in steady states of biochemical reaction systems with or without some flux rates fixed. Comput Appl Biosci 9: 79–85. PMID: 8435772
35. Srienc F, Unrean P (2010) A statistical thermodynamical interpretation of metabolism. Entropy 12: 1921–1935. doi: 10.3390/e12081921
36. Unrean P, Srienc F (2011) Metabolic networks evolve towards states of maximum entropy production. Metab Eng 13: 666–673. doi: 10.1016/j.ymben.2011.08.003 PMID: 21903175
37. Bagley RJ, Farmer JD (1992) Spontaneous emergence of a metabolism. In: Langton CG, Taylor C, Farmer JD, Rasmussen S, editors, Artificial Life II. Redwood City, CA: Addison-Wesley, pp. 93–140.
38. Pons P, Latapy M (2006) Computing communities in large networks using random walks. J Graph Algorithms Appl 10: 191–218. doi: 10.7155/jgaa.00124
